# Methodological Individualism: Still a Useful Methodology for the Social Sciences?

**DOI:** 10.1007/s11293-022-09740-x

**Published:** 2022-03-21

**Authors:** Reinhard Neck

**Affiliations:** Department of Economics and Karl Popper Foundation Klagenfurt, Alpen-Adria-Universität, Klagenfurt, Austria

**Keywords:** Methodological individualism, Critical rationalism, Social philosophy, Methodology of the social sciences, B00

## Abstract

This paper explains the role of methodological individualism as a methodology for the social sciences by briefly discussing its forerunners in economics and sociology, especially in the works of Carl Menger and Max Weber, followed by some comments on Karl Popper’s and other critical rationalists’ contributions as well as rational choice theories. Some recent arguments against methodological rationalism are then provided, including counterarguments, mainly based on exemplary work by economists and sociologists. This paper proposes a scheme for analyses using (weak) methodological individualism, in particular, arguing that evolutionary approaches to the explanation of economic and other social phenomena that accord with methodological individualism suggest that it is a successful and progressive methodology for economics and sociology.

## Variations on Individualism

The relationship between the individual and a group or society is one of the basic problems in the social sciences. In principle, this relationship can be considered in three ways. Individualism sees the individual (or the human being) as the primary unit and characterizes groups by the properties, actions, and behavior of individuals belonging to them. Holism (or collectivism) starts from a group or collective (including the state) and regards individuals as primarily or completely determined by the collective. Finally, social scientists not committed to either of the two consider the individual and the collective jointly in their interplay and at the same level of analysis.

Individualism and holism can refer to the existence and essence of individuals and a society, in particular the question as to whether a society exists independently of the individuals comprising it or not, the latter position being ontological individualism. The dictum of former UK Prime Minister Margaret Thatcher (1987, p. 30) that “There is no such thing as society” is a popular example of this attitude. Normative or moral individualism (also political individualism), on the other hand, is a normative position according to which ethical questions always refer to the behavior of and the consequences for individuals instead of addressing ethical requirements to groups, societies, or the state. Finally, methodological individualism and holism, refer to the methodology of the social sciences, and more specifically to the question as to how to explain social phenomena in the real world. Methodological individualism explains them by the behavior and actions of individuals, while methodological holism takes collectives such as society or the state as the starting point.

According to Kaushik Basu (2018, p. 1815), “Methodological individualism holds that a proper explanation of a social regularity or phenomenon is grounded in individual motivations and behaviour.” This position in the philosophy of science or methodology can be regarded as a paradigm for the social sciences in the sense of Thomas Kuhn (1962). One may distinguish between a strong (strict) definition of methodological individualism demanding explanations in terms of isolated individuals and weak (mild) definition where interactions of individuals are also included in the explanation. The former version is very rarely advocated. Within economics, the claim to provide microeconomic foundations for macroeconomic phenomena is a similar (though not identical) position, and in sociology and political science the related (and again not identical) rational choice theory follows similar methodological prescriptions. It is generally acknowledged that ontological, moral, and methodological types of individualism do not follow strictly from one other, although the motivation for all of them often stems from a general normative position of advocating the freedom of the individual, i.e., political liberalism.

This paper first sketches some historical aspects of methodological individualism, followed by a critical discussion of some of its versions. A position of weak methodological individualism is then developed from the perspective of critical rationalism, the philosophy of Sir Karl Popper. Consideration is then given to some recent arguments against methodological individualism. A scheme is then proposed showing by example how methodological individualists can structure the analysis of actions and interactions. The conclusion argues that methodological individualism, in spite of its long history, is still in the first phase of its potentialities and can be a successful and progressive research program for economics.

## Historical Developments in Methodological Individualism

The idea that social phenomena can be better explained by the behavior of individuals than without recourse to that behavior is rather old. It can already be found in Adam Smith (1776) and his idea of the invisible hand, which states that rational individuals following egoistical motivations may bring about something useful for society as a whole. This metaphor for the coordination of a multitude of special interests serving the common good is an element of methodological individualism, even though Smith did not rely exclusively on this coordination mechanism. According to Murray Rothbard (2006), Smith’s idea was already present in the writings of the late scholastic school of Salamanca, which in turn can be traced back to Aquinas and possibly to Aristotle (Höffner, 1941).

An important dispute about methodological individualism versus holism was the Battle of Methods (*Methodenstreit*) in economics between Carl Menger and Gustav Schmoller (Fusfeld, 2018; Louzek, 2011). Menger (1883), the founder of the Austrian School of Economics, argued in his methodological treatise that the rational decisions of individuals result in social outcomes that are, in general, different from what the individuals had planned. These unintended social consequences of intentional individual behavior are the most interesting topics in the social sciences in general and in economics in particular, he postulated (Neck, 2014). Schmoller (1883), the leader of the younger German Historical School of Economics, reacted to Menger’s book in a review which denied the possibility of regularities of individual behavior independent of space and time and advocated inductive empirical studies emphasizing the observation of groups and public institutions. Apart from their political positions (Menger was a moderate liberal, Schmoller a “socialist of the chair” *Kathedersozialist*), their differences over methodology resulted in a fierce debate accompanied by hostilities between German and Austrian economists which lasted until the 1920s.

The notion of methodological individualism was probably introduced into the literature on economics by Joseph Schumpeter (1908, 1909), who also wrote extensively about the idea in his history of economic theory (Schumpeter, 1954) and identified it with the Austrian School of Economics, although he himself cannot be characterized as being a devoted follower of either of them. The most influential justification of methodological individualism, however, was provided by the German sociologist and economist Max Weber (1922). He linked it to the concept of understanding (*Verstehen*), which was elaborated on by the neo-Kantian Southwest School of philosophy (especially Wilhelm Windelband and Heinrich Rickert) and was originally developed as a methodology for historical studies to understand the motivations and actions of individuals in the past. In this approach, human actions are explained by subjective intentions referring to meaningful mental states, which are only possible on an individual level and, hence, naturally confirm methodological individualism.

Weber distinguished between different types of social actions by individuals. These are broader than those defined by the neoclassical (including the Austrian) schools of economics because for Weber, rational behavior is only one possibility (besides traditional and emotional behavior), albeit important as a basic concept and standard for comparisons. In contrast to later conceptions of methodological individualism, especially those following critical rationalism, Weber advocated understanding as a separate methodology for the humanities and social sciences as opposed to explanation as the method of the natural sciences. Nowadays the explanation of real phenomena is understood as being the common aim of all sciences. The formulation and testing of hypotheses are regarded as the most prominent method, also in the social sciences, and especially in economics.

Among the followers of Menger, Ludwig (von) Mises (1963), who was also influenced by Weber, developed a rather extreme version of methodological individualism, which he called praxeology. He stated that individuals always act rationally, making this basic postulate of economics *a priori* for deriving economic laws. This idea is equivalent to the tautology that human decisions in a given situation attempt to achieve the best possible result given their information and the circumstances of the situation as they see it. Friedrich (von) Hayek, who was strongly influenced by Mises, followed this apriorism until he became acquainted with Popper’s philosophy of science. Hayek’s main contribution to methodological individualism is his concept of a spontaneous order, meaning an economic order not designed by a plan but developed out of the interactions of rational individuals and clearly a derivative of Menger’s ideas. According to Hayek, methodological individualism contributes to a more modest attitude in social scientists by pointing towards the limits of reason and an avoidance of scientism.

## Critical Rationalism and Methodological Individualism

Current debates on methodological individualism usually start from the ideas of Karl Popper and his followers. Popper (1945, 1957), who was influenced by Hayek, extended the concept to political philosophy and linked it to the methodological proposal of situational analysis (Popper, 1967). With the methods of critical examination and falsification of theories as its cornerstones, Popper applied his philosophy of science to the social sciences as well, which he did not consider to be fundamentally different from the natural sciences. In contrast to Max Weber, he saw *Verstehen* and hermeneutics not as a method in its own right but as a possible way of explaining empirical and especially historical observations. Popper’s situational analysis is his method for the social sciences, which is an explanation of the decision situation of agents within a social context, either historical or current. Although Popper sees this method as a transfer from the methodology of neoclassical economics to the social sciences in general, it can be argued (Palacio-Vera, 2020) that it is rather a pattern of explanation taken from the historical sciences.

Popper’s motivation for developing his version of methodological individualism was his fervent opposition to the historical philosophies of Hegel and Marx, which contained a teleological view of history. These philosophies, which Popper called historicism, assumed a goal in the development of human society towards which that development must, of necessity, converge. The task of social science, according to these philosophies, was to predict the future development of society and support policies toward that development. Popper showed that historicism-based philosophies led to totalitarian societies like those of fascism, national socialism, and Marxist-Leninist communism.

In opposition to these positions and their terrible consequences in the twentieth century, Popper developed his idea of an open society, which allowed for individual freedom through uninhibited criticism and discussion. Behind this idea lies a version of moral individualism, demanding that individuals should be able to enjoy the greatest possible freedom consistent with the same freedom for others, which he summarized in the oft quoted principle “I may be wrong and you may be right, and by an effort, we may get nearer to the truth.” (Popper, 1945, vol. 2, p. 225).

It may be noted that a similar theory of a free society was developed by the logician and Catholic monk Joseph M. Bochenski (1974, 1986) from his theory of authority and the distinction between epistemic and deontic authority, which are based upon knowledge and right of command respectively. Although Bochenski provides a logical analysis of the concept of a free society, his argument is essentially a moral one: His theorems 3.6 and 3.7 (Bochenski, 1974, p. 43f.), suggest that each human being is an (epistemic) authority for all other humans in at least one field (e.g., the person’s own pain, such as a toothache) but no human being is an authority in all fields for any other human (and, *a fortiori*, for all humans). These theorems are based on pure logic plus the idea of individuals with a minimum amount of knowledge. However, the requirement that, in a free society, epistemic and deontic authority should overlap as much as possible is clearly a normative requirement for a free or open society. With both Popper and Bochenski, moral arguments are behind the decision to favor methodological individualism. Although the connection between the two versions of individualism is not one of necessity, for these and other philosophers it provides a strong motivation.

Popper and Hayek had similar views about methodological individualism and the limits of holistic attempts to plan for a nation or a society, although they differed in the detail, with Hayek being more skeptical about the possibility of economic reforms being introduced by a government. A more systematic approach to the concept of methodological individualism was elaborated on by Popper’s former student and later colleague J.W.N. Watkins (1952a, b, 1955, 1957, 1958, 1959a, b). He rejected methodological holism by explicitly pointing out that there are no laws for a social system as an organic whole from which the behavior of its components could be derived. Explanations of social phenomena that do not recur on the motivations and actions of individuals are not rock-bottom explanations according to Watkins. They have a distinctly lower status than methodologically individualist ones, being at best provisional.

Interestingly, in 1957, Watkins presented the trade-off between inflation and unemployment in the Phillips curve as an example for this position, which was later the starting point for the break-up of the post-WWII Keynesian Consensus and the development of the monetarist and new classical alternatives with their emphasis on the microeconomic foundations of macroeconomics. Methodological individualism can, hence, be regarded as a successful paradigm, at least in macroeconomics.

Critiques of the critical-rationalist version of methodological individualism, mostly by other philosophers, and some replies to them are collected in O’Neill (1973); see also Udehn (2001). Later critics (Basu, 2018; Bhargava, 1992), argued against methodological individualism by stating that individuals’ decisions are made within a context in which essentially social concepts are important, and these cannot be reduced to individual concepts. Arrow (1994) also used this argument when stating that prices are essentially social phenomena. This argument ignores the fact that in neoclassical microeconomic theory (to which Arrow himself so profoundly contributed) these prices are explained by the interactions of individual agents on the demand and supply sides. There is no group or supra-individual authority responsible for the prices in a market economy. Rather there are the competitive endeavors of thousands of individual market participants. Hence, neoclassical theory for that matter is fully compatible with (at least, a weak version of) methodological individualism.

Further discussion within the camp of critical rationalism changed the theses originally advocated by Popper and Watkins to some extent, with both of them modifying (at least tacitly) their original positions. In particular, Popper’s concept of a “World 3” of products of human thought such as ideas, theories, works of art, values, institutions, and traditions, which, although man-made, have influences independent of those creating them, is apparently in the tradition of holistic arguments. Although its characterization as a social ontology is not justified, the purported influence of World 3 on individuals other than their generators needs some clarification by methodological individualists, such as stressing that these influences only occur through active acquisition by the influenced individuals, who always have the ability to discard them, as the example of reintegrated criminals shows.

**Fig. 1:**
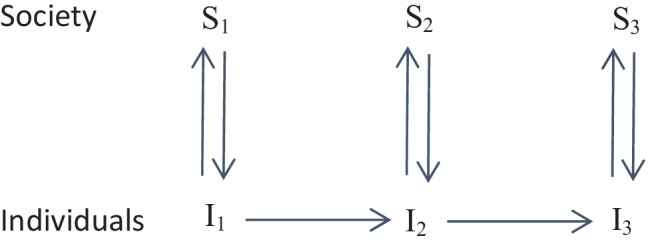
Popper’s institutional individualism according to Udehn (2001)

Another branch of critical-rationalist methodological individualism is institutional individualism as proposed by Agassi (1960, 1975) and partly by Popper himself in his later work. In this variant, institutions exert some influence upon individual decisions, as in Popper’s World 3 (although it precedes the latter). According to Udehn, this version is incompatible with the methodological individualism as defined by the Austrian School and is expressed diagrammatically as shown in Fig. 1 (Udehn, 2001, p. 227).

Udehn’s criticism is not entirely justified. Indeed, Menger had set himself the explicit aim of explaining the spontaneous emergence of institutions and provided such an analysis for the institution of money. It is also true here that the institutions, once created, exert an influence on later generations of individuals. However, the same argument in the preceding paragraph justifies the use of the label of (weak) methodological individualism for this approach.

## Further Applications of and Controversies Surrounding Methodological Individualism

On the one hand, methodological individualism has many applications as a methodology in several social sciences. It is not confined to economics, where it was and still is the dominant approach, at least nominally. However, prominent sociologists and political scientists adopted and propagated it in their disciplines as well. Examples of the former are sociologists James Coleman (1986, 1990) in the U.S., Raymond Boudon (1974) in France, and Hartmut Esser (1999, 1999–2001) in Germany. Even more proponents of methodological individualism, mostly in the form of the rational-choice approach, can be found leading political science journals, such as the *American Journal of Political Science*, the *American Political Science Review*, or *Comparative Political Studies*. The wide dissemination of methodological individualism among political scientists may be explained by the intrusion of public choice into the fields of political theory and empirical analysis. Conferences and journals focused on political economy (as used in economics, i.e., public choice) are open to scholars from political science departments and are frequently used as outlets for their research. Even the concepts and theories of Karl Marx, who is generally regarded as a collectivist (or holist) *par excellence*, have been investigated from a methodologically individualist perspective (Elster, 1982, 1985).

An overview of the broad applicability of the rational-choice approach is given by Gebhard Kirchgässner (2010), who also discussed the reasons for its success. One of them is the use of relatively sophisticated mathematics, which permits precise formulations of problems, theoretical assumptions, and results. Somewhat ironically, after the Great Recession and in the Covid-19 pandemic crisis, those very traits of the economic approach in general and of methodological individualism in particular were being increasingly criticized by heterodox economists claiming that their relevance in and applicability to the real world are being sacrificed for the increase in precision. They accused economists of economic imperialism and advised them to learn from the methods and results of sociology and political science instead. Although learning from related disciplines is certainly a good idea, it would not be wise to forego human capital invested in developing and using techniques within one’s own discipline. Instead, following the research strategy proposed by Nobel Laureate Leo Hurwicz (1973) is recommended which is to investigate problems of institutions (and more generally of social phenomena) by using analytically advanced methods.

With respect to methodological individualism, philosophers (and, to a lesser extent, sociologists) have investigated the relative merits of methodological individualism and holism as research strategies. Overviews of relevant philosophical arguments are given by Heath (2020) and Zahle (2021). See also Zahle and Kincaid (2019), Kincaid (1996, 1997, 1998, 2004, 2018), and the contributions in Zahle and Collin (2014). These authors are more critical of the individualist methodology than most practicing economists. Lack of space precludes a detailed analysis of all objections to methodological individualism raised in this literature; hence only a few remarks are included herein.

Mandelbaum (1955) asserted that there are social concepts which cannot be reduced to descriptions of individuals. The much-cited example of a bank teller was used to support this position as the notion of a bank, according to him, is one such social concept and a holistic explanation is therefore required. However, banks like other institutions, such as money, can be explained by their emergence from interactions between agents who preferred to organize the lender-borrower relationship in a more efficient way. That these institutions existed beyond the lifetime of the original generation of bank creators is because later generations shared the same desire as the original ones, i.e., they preferred using banks. That banks cease to work in certain situations, such as runs on them, shows that an explanation of their continued existence requires an individualistic explanation based on the continuing trust of the lenders and borrowers, which is just what an individualist methodology would do.

Political scientists List and Spiekermann (2013) entered the debate about supervenience and realization by claiming that some supervenient social properties are causally effective for the explanation of social processes. Their example of the failure of the Copenhagen climate summit in 2010 due to (among other structural facts) the lack of common interest among participants, who were not fully aware of the lack of commonality, is taken as evidence of the need for a holistic explanation. Even if the different interests had been recognized, the social property of the summit failing would still have occurred. However, it is this very lack of a common interest which points to the need to consider the preferences of the individual participants, irrespective of whether they were aware of these preferences or not. This is a strong argument for methodological individualism by pointing towards the properties of those individuals, in contrast to the claims of List and Spiekermann (2013).

Zahle’s (2021) examples of cases for which she claimed purely holistic explanations were required do not work either. Misleading is the suggestion that the French Revolution occurred in 1789 instead of in 1750, for example, caused by the weak economy of France in 1789 due to the previous use of resources in the American War of Independence and increased competition with Great Britain. These events can only be explained by referring to the decisions of the individuals governing in France (mainly the king and his advisors in the absolutist system) and not by some social entity. They were man made, as high sovereign debt crises generally are. Thus, resorting to a holistic explanation is superficial.

Her other argument for the requirement of a holistic explanation was that the connection between an increase in the money supply leading to an increase in price level may be the result of different individual-level mechanisms, which, therefore, should not be invoked to explain the law the quantity theory of inflation purports to hold. However, this shows that the quantity theory is incomplete and does not hold generally, as has been confirmed by empirical tests of that theory. The reason for this is that there is no such law at the purely social level. Rather an adequate theory of inflation needs additional, individual-level explanatory elements, thus providing a case for (instead of against) methodological individualism.

## A Proposal

**Fig. 2:**
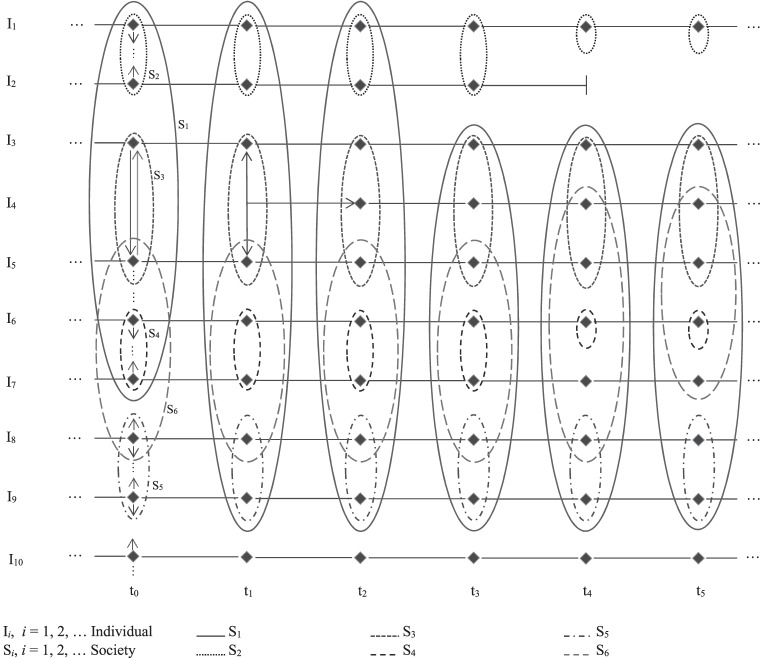
An example of a scheme for weak methodological individualism

In view of the theoretical arguments presented herein, I now propose, by example, an analytic scheme in the spirit of weak methodological individualism. The example in Fig. 2 illustrates the basic idea.

Individuals are denoted by I_*i*_, *i* = 1, 2, …, along a time line t_*k*,_ *k* = 0, 1, 2…, Societies, which are (formal or informal) groups (including institutions) encircling the individuals who are their members, are denoted by S_*i*_. Note that any individual is a member of no, one, or more societies and may switch (leave and/or enter) membership at any time point t_*k*,_ where each time point denotes a situation where at least one individual switches. It is important to note that such a switch is possible (although probably at some cost to the switching individual, when their ties to the other members of the group are strong) for each individual, which assumes a certain degree of individual freedom prevailing within the group (society) concerned. Interactions between individuals within a society or across different societies are denoted by arrows for the individuals involved; they are omitted for later time points in Fig. 2 to simplify the picture. Arrows in both directions denote two-way interactions, which may be characterized by cooperation, or conflict, or both. An arrow with arrowheads at both ends denotes especially intensive, cooperative interactions. For example, Individuals I_3_ and I_5_ interact intensively at time point t_1_, giving birth to Individual I_4_ at time point t_2,_ who is then part of their society (family) S_3_. Individual I_1_ leaves society S_1_, together with individual I_2_, at time point t_2_ and leaves society S_2_ at time point t_3_, staying alone afterwards. Individual I_2_ dies at time point t_3_. Similar interpretations may be given for other individuals such as I_6_, for example.

The purpose of this example is to show how, from the position of weak methodological individualism, the actions and interactions of individuals as well as their membership in different groups can be analyzed. For example, the interactions may be investigated using game theory if the focus is on strategies when conflicts and/or possibilities for cooperation are present, using sociographic analysis for small groups, or using management and organization theories if the focus is on the internal relations within such organizations.

The individualistic feature of the scheme is reflected in the primary role of the individuals as sole agents. No society acts there (there are no arrows from or into societies). The actions of groups can be analyzed by considering the internal decision processes within the specific group alone. This makes sense in various circumstances. For example, the decisions of a government, at least in a public-choice analysis, should be investigated by considering the process by which the ministers and members of different groups influencing them interact until some group decision is agreed upon, which, in turn, must be carried out by one or several individuals (ministers and their bureaucrats). The advantage of such an analysis is the potential identification of the individual interests of the agents and relating the group decision to them instead of working with the fiction of a common interest. If it actually exists in the first place, the latter is the interest of several (or rarely all) individuals participating in the decision (and only sometimes of agents not participating in it as well).

The weakly individualistic feature of the scheme in terms of explanatory individualism comes from the tolerance toward holistic approaches as first-order approximations to a social science analysis. In some cases, a truly individualistic analysis may not be required, for example, when a government has to decide on measures to take in an emergency situation, such as the Covid-19 pandemic. Then a small group, or even one person, has to decide what to do. No analysis of the internal decision-making process of the group may be required. Another case is the analysis of macroeconomic hypotheses for forecasting purposes where considering every single individual will be too time consuming or not feasible at all. The first generation of Keynesian macroeconomic models provides examples for this case. Uncovering the neglected microeconomic foundations in these models led to improvement, for instance in the dynamic stochastic general equilibrium (DSGE) and New Keynesian models, although these are still imperfect as they rely on the assumption of the representative individual, which for many issues (like distributional questions) is highly inadequate (Kirman, 1992). Relaxing other assumptions of these models, such as the rational-expectations assumption, could lead to further convergence to the real world and, hence, improve the explanatory power of macroeconomic models (e.g., De Grauwe & Ji, 2019).

It is not intended to use the scheme presented earlier as an element of an ontology, arguing that the world is as depicted there. Apart from the fact that ontological issues are mostly metaphysical and cannot easily be made the subject of scientific analysis, here the scheme is proposed as a (weakly) individualistic way of explaining social phenomena. However, what is claimed here, is that it provides a more appropriate method for analyzing social science problems than holistic approaches that do not consider the actions and interactions of individuals.

## Concluding Remarks

In this paper, the origins and historical development of methodological individualism were described, with particular emphasis on the contribution of critical rationalism to that concept. The impact of methodological individualism on social sciences other than economics, from which it started, was discussed. Some arguments in favor of the necessity of holistic explanations were rejected. It must be admitted that methodological individualism is a malleable concept to be applied in a tolerant way concerning the admissibility of including some seemingly holistic elements in explanations, such as institutions. However, the claim holds in these cases as well that an individualistic methodology can yield crucial insights. The strict version of methodological individualism, which only considers the isolated actions of individuals, is not followed by a substantial number of authors. Instead, one must turn to a weak version. For this, a scheme was proposed as a blueprint for analyzing social situations and processes. Methodological individualism is a progressive research strategy that is especially suitable for a globalized open society where traditional links and group loyalties are weak and individuals can exercise liberties to follow their own dispositions and desires.
